# Psychosocial Predictors of Ventilator weaning Outcomes among patients in intensive care units

**DOI:** 10.1016/j.heliyon.2024.e24385

**Published:** 2024-01-19

**Authors:** Mohamed Gamal Elsehrawy, Ahmad M. Saleh

**Affiliations:** a Department of Nursing Administration and Education, Prince Sattam Bin Abdulaziz University, College of Nursing, Kingdom of Saudi Arabia; b Nursing Administration, Nursing Faculty, Port-Said University, Egypt

**Keywords:** Intensive care units, Psychosocial predictors, Ventilator weaning & weaning outcomes

## Abstract

**Background:**

Ventilator weaning is a process of discontinuing mechanical ventilation and transitioning patients to independent breathing after a period of mechanical support. Weaning outcomes among the critically ill in intensive care units (ICUs) vary significantly among individuals, leading to considerable variation in healthcare costs, length of hospital stay, morbidity and mortality. Addressing psychosocial aspects of care can improve weaning outcomes.

**Objective:**

This study aimed to determine the effect of psychosocial factors (social support, family/significant other support, ability to communicate) on weaning outcome within intensive care patients. This research hypothesized that psychological and social factors play a role in determining ventilator weaning outcomes among ICU patients.

**Methods:**

This study used a longitudinal, retrospective research design to analyze positive and negative psychosocial predictors of ventilator weaning outcomes. Data collection methods include interviews and questionnaires with patients and their families, as well as clinical data from the patient's medical records.

**Results:**

presence of anxiety, depression, and hallucinations, have a negative relationship with weaning outcomes regarding a patient's psychological characteristics (r_s_ = −0.207, −0.163, −0.158), while communication with a patient during mechanical ventilation have a positive relationship with weaning outcomes regarding a patient's psychological characteristics (r_s_ = 0.152; p ≤ 0.05). Moreover patients who have fear during weaning trials, feeling neglected, and feeling insecurity have a negative relationship with weaning outcomes while gaining family support during mechanical ventilation have a positive relationship with weaning outcomes (rs = 0.144; p ≤ 0.05).

Significance of results, the findings suggests that psychosocial factors, such as anxiety, depression, patient's perception of their illness, motivation to wean, and family involvement can influence the success of ventilator weaning among patients in ICUs.

## 1 Introduction

One of the most frequently used therapeutic modalities in the critical care unit is mechanical ventilation (MV). In the last two decades, significant effort has been made to determine the best method for weaning patients off MV in various ICU systems and structures [1]. The core purpose of mechanical ventilation is to help exchanges of gases until the patient's breathing problem is resolved. The methods used to carry out oxygenation are a variety of cutting-edge tools, models, and procedures. These methods primarily help patients initiate ventilation and partially or fully support their breathing. Despite the availability of such cutting-edge equipment, mechanical ventilation is linked to a variety of patient physiological and psychosocial complications [2].

Latest studies have signposted that problems resulted from the attachment with mechanical ventilation reduced when the length of stay is condensed [3]. A lot of social and psychiatric problems that may occur like stress, anxiety, sensory deprivation, and increasing length of hospital stay [4]. Ventilator weaning is a complex process that requires the coordination of several physiological and psychological factors, including the patient's ability to tolerate spontaneous breathing, their cognitive and emotional functioning, and their support system [5].

Although MV frequently saves lives, it can also cause serious, even fatal, problems for the patients [6]. The diagnosis of the patient's preparedness for weaning is one of the important tasks that nurses perform in the ICU. Effective weaning features include better weaning preparation interventions, regular weaning readiness assessments, techniques to enhance and encourage spontaneous breathing during weaning, and the use of spontaneous breathing trials (SBT) to gauge the likelihood of weaning the patient from the ventilator [7].

After being detached from a mechanical ventilator, some patients have reported experiencing psychiatric issues like amnesia, trouble focusing, and delusion. However, these complications happen less frequently if the patient is adequately supported while using the ventilator. Despite the fact that there have been many studies on mechanical ventilation, it seems essential to carry out a study that focuses solely on the psychological experiences of the patients who are using it, so that their psychological needs can be met to stop any subsequent issues [8].

While emotional stress and psychological complications from weaning still affect patient years after discharge and have an impact on their health, the experiences and psychological needs of these patients have not yet been adequately explained for the ICU. The previous studies ‘ambiguity regarding clinicians’ and family members’ experience with the termination of ventilator support is further emphasized. The process of withdrawing life-sustaining measures, including the cessation of mechanical ventilation, has been a topic of debate for ICU clinicians that have persisted over time [9]. Although the success of ventilator weaning is strongly correlated with the severity of the illness or injury, psychological variables have been linked to weaning outcomes but are still largely unexplored. Therefore, the goal of the current study is to assess psychosocial predictors of ventilator weaning outcomes among patients of critical care units [8].

Psychological factors can influence the success of ventilator weaning. Patients who are anxious or stressed may have increased physiological responses, such as elevated heart rate and respiratory rate, which can complicate the weaning process. Addressing psychological factors may contribute to a smoother weaning process and increase the likelihood of successful extubation [6]. In addition, prolonged mechanical ventilation experiences can contribute to the development of Post-Traumatic Stress Disorder in some patients. Understanding and managing psychological factors during ventilator weaning may help prevent or mitigate the long-term psychological impact on patients [4].

### 1.1 Significance of the study

The transition from complete ventilatory assistance to spontaneous breathing occurs during the process of weaning from mechanical ventilation. Approximately 70 % of intubated mechanically ventilated patients are extubated on the first try at spontaneous breathing (SBT), either by disconnecting from the ventilator or after breathing for brief amounts of time (30–120 min) with low levels of pressure support. The surviving patients (roughly 30 %) require gradual withdrawal from artificial ventilatory support. Although there is individual variation in weaning success and the degree of experienced distress, the weaning process is psychologically taxing for all patients. Prolonged mechanical ventilation and the uncertainty surrounding weaning can lead to psychological distress, moreover patients experiencing high levels of stress or anxiety may require a longer time on the ventilator, leading to increased healthcare costs and potential complications associated with prolonged mechanical ventilation. The psychosocial predictors of ventilator weaning outcomes in patients have not been systematically evaluated [7].

### 1.2 Specific aims

To determine the effect of psychosocial factors (social support, family/significant other support, ability to communicate) on weaning outcome within intensive care patients. This could be achieved by assessing the psychological problems and characteristics among mechanical ventilated patients and it's impact on successful weaning.

## 2 Methodology

### 2.1 Design

This study was use the retrospective descriptive comparative study using a medical records review. Patient data abstracted from medical records following hospital discharge. ICU patients who required mechanical ventilation during their hospital stay comprised the sample.

### 2.2 Subjects

Study participants will consist of patients who received mechanical ventilation in each of the two units, MICU and SICU during the period January 1, 2021 through January 1, 2022. Convenient sample of patients was selected to complete the data collection, the total patients participated in the study was 247 from intensive care units during the time of data collection. Adult patients included when they received mechanical ventilation for a minimum of 24 h. Patients excluded only if their age is less than 18 years, with irreversible neuromuscular diseases and dependent on MV, or if the patients were determined to be terminal upon admission to the intensive care unit such as brain death (organ donor awaiting organ harvest), cancer metastasis, intubated because of lung cancer and multiple organ failure.

Being on a mechanical ventilator for at least 24 h while using a tracheostomy or endotracheal tube, being a male or female over the age of 18, and being able to provide data are the inclusion requirements. While Patients who are brought to the ICU but are not receiving mechanical ventilation, patients who are disoriented, patients who are under the age of 18, and patients who cannot speak Arabic are excluded.

### 2.3 Measures

A medical records data abstraction data collection form, was used as a guide for data collection. This form was devised for this investigation and based on the specific aims of the study. Sections of the form include: Demographic data, psychosocial indices and predictors. Patient record examined to look for data and information regarding psychological state before patient admitted to ICU as (marital state, economic state, and psychological problems), also data occurring after weaning and evaluation of general condition of patients. Social state also examined before attaching patients with mechanical ventilation and in the weaning period followed by patient's condition after weaning from mechanical ventilation and patient outcome. Data also collected from patients using structured interview method after three days of weaning of M.V., this interview have questions related to anxiety, depression, fear, and difficulties occurred during and prior to weaning, this interview occurred after obtaining permissions with informed consent to patients and their families.

### 2.4 Ethical consideration

The study was approved by the Institutional Review Board (IRB), it reviewed for ethical approval for the research proposal with approval number SCBR-045-2022. Informed consent both oral and written were obtained from eligible patients and their first-degree families. Patients and their families were informed of the purpose and nature of the study, as well as any potential risks or drawbacks of the data collection process. They were also made aware of their right to withdraw from the study at any time and without cause, and that we would accept their withdrawal if they did so.

### 2.5 Data analysis

Statistical analysis and calculation was performed using SPSS statistical package, version 22.0. Sample characteristics were analyzed using descriptive statistics. Comparison between groups was analyzed by using chi-square test for categorical data. Wilcoxon signed rank test was used for comparing two groups in the weaning outcome. Logistic regression was utilized to identify factors that predicted weaning success. Spearman correlation test was used for testing the correlation between psychological problems with the weaning outcome.

## 3 Results

The first table showed that there was a statistically significant difference in weaning outcomes about patient's socio-demographic characteristics between different age groups, (X^2^) = 22.785, p ≤ 0.05, with a high percentage score of 46.3 % for 50–60 years, 27.2 % for 50–60 years and 16.9 % for less than 50 years. On the other hand, there were no statistically significant differences in weaning outcomes between the marital state and level of the economic state respectively [Table 1].

**Table 1:** Weaning outcome regarding patient's socio-demographic characteristics (n = 247).

Dimensions	Successful weaning		Failed weaning		X^2^	P value
	N.	%	N.	%		
Age						
Less than 50 year	23	16.9	3	2.7	22.785	0.001*
50: <60 years	63	46.3	39	35.1		
60: <70 years	37	27.2	45	40.5		
70 years and more	13	9.6	24	21.6		
Gender						
Male	82	60.3	56	50.5	2.402	0.125
Female	54	39.7	55	49.5		
Marital state						
Married	97	75.8	87	73.1	4.975	0.174
Divorced	8	6.3	12	10.1		
Widowed	5	3.9	10	8.4		
Single	18	14.1	10	8.4		
Level of economic state						
Low	14	10.9	13	10.9	0.261	0.878
Moderate	62	48.4	54	45.4		
High	52	40.6	52	43.7		
Previous history of mechanical ventilation						
Yes	47	36.7	49	41.2	0.516	0.473
No	81	63.3	70	58.8		

X^2^ = Chi Square test.

p: p value for associating between different categories *: Statistically significant at p ≤ 0.05.

The second table presented statistically significant differences in weaning outcomes for individuals with a presence of anxiety, presence of depression, presence of hallucinations, and communication with a patient during mechanical ventilation than those who did not (Z = 10.583, p ≤ 0.05), (Z = 6.542, p ≤ 0.05), (6.201, p ≤ 0.05), (Z = 6.684, p ≤ 0.05) respectively. The Spearman correlation coefficient test indicates a negative relationship r_s_ = 0.207-, 0.163-, 0.158-; accordingly, Except, for communication with a patient during mechanical ventilation which have a positive relationship with weaning outcomes regarding a patient's psychological characteristics r_s_ = 0.152; p ≤ 0.05 [Table 2].

**Table 2:** Comparison of weaning outcome regarding patient's psychological characteristics (n = 247).

Dimensions	Successful weaning		Failed weaning		Z	P value
	N.	%	N.	%		
Presence of anxiety prior to mechanical ventilation						
Yes	97	39.3	98	39.7	10.583 **(-0.207-)**	0.002*
No	39	15.8	13	5.3		
Presence of depression prior to mechanical ventilation						
Yes	92	37.2	91	36.8	6.542 **(-0.163-)**	0.011*
No	44	17.8	20	8.1		
Presence of delirium prior to mechanical ventilation						
Yes	62	25.1	39	15.8	2.763	0.096
No	74	30.0	72	29.1		
Sleep quality prior to mechanical ventilation						
Bad	81	32.8	57	23.1	1.674	0.433
Sometimes good	39	15.8	38	15.4		
Good	16	6.5	16	6.5		
Communication with patient during mechanical ventilation						
Yes	39	15.8	48	19.4	6.684 **(0.152)**	0.017*
No	97	39.3	63	25.5		
Presence of hallucinations prior to mechanical ventilation						
Yes	68	27.5	73	29.6	6.201 **(-0.158-)**	0.013*
No	68	27.5	38	15.4		

z: Wilcoxon signed rank test p: p value for associating between different categories *: Statistically significant at p ≤ 0.05.

(r) for spearman correlation test.

Moreover the third table showed that there were statistically significant differences in psychological problems during weaning for individuals who have fear during weaning trials, feeling neglected, feeling insecurity, and gain family support during mechanical ventilation than those who did not have (Z = 3.992, p ≤ 0.05), (Z = 5.223, p ≤ 0.05), (10.447, p ≤ 0.05), (Z = 5.134, p ≤ 0.05) respectively. The Spearman correlation coefficient test indicated a negative relationship rs = 0.127-, 0.145-, 0.206-; accordingly, except, for patients gaining family support during mechanical ventilation which have a positive relationship with weaning outcomes (rs = 0.144; p ≤ 0.05) [Table 3].

**Table 3:** Psychological problems during weaning among mechanical ventilated patient's (n = 247).

Dimensions	Successful weaning		Failed weaning		Z (r)	P value
	N.	%	N.	%		
Fear during weaning trials						
Yes	91	36.8	87	35.2	3.992 **(-0.127-)**	0.046*
No	45	18.2	24	9.7		
Getting bored						
Yes	83	33.6	75	30.4	1.134	0.287
No	53	21.5	36	14.6		
Gain family support during mechanical ventilation						
Yes	93	37.7	90	36.4	5.134 **(0.144)**	0.023*
No	43	17.4	21	8.5		
Pain						
Yes	81	32.8	68	27.5	0.074	0.786
No	55	22.3	43	17.4		
Agitation						
Yes	42	17.0	40	16.2	0.732	0.392
No	94	38.1	71	28.7		
Discomfort because of endotracheal tube connection						
Yes	84	34.0	78	31.6	1.959	0.162
No	52	21.1	33	13.4		
Noise						
Yes	76	30.8	56	22.7	0.725	0.395
No	60	24.3	55	22.3		
Insomnia						
Yes	79	32.0	68	27.5	0.255	0.613
No	57	23.1	43	17.4		
Inability to talk						
Yes	87	35.2	74	30.0	0.196	0.658
No	49	19.8	37	15.0		
Loneliness						
Yes	69	27.9	67	27.1	2.288	0.130
No	67	27.1	44	17.8		
Feeling isolated						
Yes	65	26.3	65	26.3	2.841	0.092
No	71	28.7	46	18.6		
Feeling neglected						
Yes	75	30.4	77	31.2	5.223 **(-0.145-)**	0.022*
No	61	24.7	34	13.8		
Feeling insecurity						
Yes	64	25.9	75	30.4	10.447 **(-0.206-)**	0.001*
No	72	29.1	36	14.6		

z: Wilcoxon signed rank test p: p value for associating between different categories *: Statistically significant at p ≤ 0.05.

(r) for spearman correlation test.

Finally the fourth table showed multivariate analysis, age (p < 0.05) and marital status (p < 0.05) were found to be independent predictors of successful weaning. These two predictors accounted for 67 % (R2 = 0.67) of the variance in the predicted values for successful weaning among mechanically ventilated patients [Table 4].

**Table 4:** Predictors of successful weaning among mechanical ventilated patients.

Model	Unstandardized Coefficients		Standardized Coefficients	t	Sig.	95.0 % Confidence Interval for B	
	B	Std. Error	Beta			Lower Bound	Upper Bound
(Constant)	.550	.620		.888	.376	−.671-	1.772
Age	.175	.044	.262	4.022	.000	.089	.261
Gender	.026	.075	.022	.348	.728	−.122-	.174
Marital status	.159	.036	.281	4.464	.000	.089	.230
Level of economic state	−.063-	.056	−.071-	−1.119-	.264	−.173-	.048
Presence of anxiety	.034	.104	.024	.328	.743	−.172-	.240
Presence of depression	−.010-	.091	−.008-	−.113-	.910	−.190-	.169
Presence of delirium	−.110-	.081	−.093-	−1.363-	.174	−.270-	.049
Sleep quality	.080	.051	.097	1.581	.115	−.020-	.180
Communication with patient	.100	.079	.082	1.265	.207	−.056-	.256
Fear	.036	.082	.028	.437	.662	−.125-	.197
Bored	−.133-	.077	−.110-	−1.742-	.083	−.285-	.018
Family support	−.023-	.085	−.017-	−.268-	.789	−.190-	.145
Pain	−.063-	.073	−.053-	−.863-	.389	−.208-	.081
Agitation	.053	.077	.043	.689	.492	−.099-	.206
Discomfort because of endotracheal tube	.100	.079	.082	1.267	.206	−.056-	.257
Inability to talk	−.034-	.086	−.028-	−.390-	.697	−.204-	.137
Loneliness	.009	.074	.008	.126	.900	−.136-	.155
Hallucinations	.097	.073	.082	1.316	.190	−.048-	.241
Feeling Neglected	−.085-	.076	−.071-	−1.122-	.263	−.235-	.065
Feeling Isolated	−.042-	.079	−.036-	−.531-	.596	−.197-	.113
Feeling insecurity	.087	.076	.074	1.147	.253	−.063-	.237

R-square = 0.667 model ANOVA; F = 2.411 * p = 0.001* Significant p < 0.05.

## 4 Discussion

The aim of the current study was to determine the effect of psychosocial factors (social support, family/significant other support, ability to communicate) on weaning outcome within intensive care patients. Findings of the current study showed that, there was a statistically significant difference in weaning outcomes about patient's socio-demographic characteristics between different age groups.

In a study found that age and weaning failure was linked. Weaning failure rates were 27.8 % in patients over the age of 80 and 22.1 % in patients under the age of 60. The function of the lungs was known to decline over time [10]. Changes in pulmonary function result from physiological changes in the parenchyma and chest wall that reduce static elastic recoil, chest wall compliance, and the power of the respiratory muscles [11]. The results of this research refute the claim that elderly patients experience weaning failure at a higher rate than adults [12].

Furthermore, presence of anxiety, depression, hallucinations, and communication with a patient during mechanical ventilation were statistically significant differences in weaning outcomes. While communication with a patient during mechanical ventilation had positive correlation with weaning outcome, but the presence of anxiety, depression, and hallucinations had negative relationship with the weaning outcomes. These findings indicate that patients with anxiety, depression, and hallucinations may have poor consequences in weaning outcome. Moreover communications with a patient during mechanical ventilation benefit the patient during the weaning process.

Patient anxiety causes them to breathe more frequently, have smaller tidal and minute volumes, and require more sedatives [13]. Because they are actively treated, receive few visitors, and are in a strange and loud environment, the majority of mechanically ventilated patients go through a lot of worry and stress, which can make them reliant on mechanical ventilation [14]. Therefore, it is very important to think about methods of relieving anxiety for ICU patients to get successful weaning process, such of these methods is the presence of the family at the patient's bedside [15].

Furthermore, a study was done on patients who underwent mechanical ventilator weaning revealed that depressive disorders can increase the possibility of weaning failure by up to three times, and increase patient distress during mechanical ventilator weaning [16]. The patients on ventilator reported psychological disorders as helplessness, passivity, hallucinations, and nightmares [17]. It was crucial to communicate with the patient throughout the weaning procedure. Continuous dialogue with the patient aided in the weaning off from mechanical ventilation. On the other hand, ineffective dialogue made the patient anxious, which slowed their recovery and lengthened their time on mechanical ventilation [18].

Study findings revealed that some psychological problems had a statistically significant difference with weaning outcome as patients with fear during weaning trials, feeling neglected, feeling insecurity, and gain family support during mechanical ventilation. The weaning outcome results was worsen in patients with fear during weaning trials, feeling neglected, and feeling insecurity, while weaning outcome was improved with gaining family support during mechanical ventilation.

According to a study indicated that more than third of participants said they felt most afraid right after the weaning trial. The weaning trial was unsuccessful for more than two thirds of mechanical ventilator patients who reported fear [19]. In addition, patients feel frustration and insecurity when a nurse does not understand them or is ignoring them at the first hours of weaning trials which cause dissatisfaction [15].

The review of the literature demonstrates that nurses are crucial in enhancing patient communication and foreseeing their requirements. To provide holistic care, it is crucial to provide physical, psychological, and technological treatment. Consequently, studies are required to better patient treatment and communication, as well as to lower patients' anxiety and stress. Training, instruction, and professional practice are therefore prioritized [20].

The findings of multivariate analysis revealed that age and marital status were found to be independent predictors of successful weaning. These two predictors accounted for 67 % of the variance in the predicted values for successful weaning among mechanically ventilated patients. These results may be attributed to patients with high age decrease their ability to have successful weaning, also patients who had a partner (spouse or wife) may communicate with him and this increase the ability to recover successfully.

Studies have shown that high age is considered to be a significant risk factor in the intensive care unit, but it is unclear how it affects weaning failure [21]. Successful weaning for patients on prolonged mechanical ventilation (PMV; >21 days) has been ascribed to a number of variables. Younger age and female gender were found to be factors that substantially predict successful weaning by univariate logistic regression analysis [22].

Moreover, another study found that, a binary logistic regression analysis was used to model the predictors for failed weaning, which were taken from the patients' clinical characteristics, comorbidities, respiratory indices, and the outcome of the first SBT. The remaining variables were added to the model using a P value-based forward selection at the alpha level of 5 % after age and gender were compelled into the model [23].

## 5 Conclusion

Based on the findings of the current study, it can be inferred that patients with anxiety, depression, or hallucinations prior to mechanical ventilation may had unsuccessful weaning while communication with a patient on mechanical ventilation may lead to Successful weaning. Those patients who experience fear during weaning trials, feel neglected, and feel insecure, had negative correlated with weaning outcome. Moreover patients were receiving family support during mechanical ventilation had positive results in weaning outcome. Age and marital status can be considered independent predictors for successful weaning outcomes. Finally, collaboration between healthcare professionals, including psychologists or mental health specialists and staff nurses with patient's families, can enhance ventilator weaning outcomes.

This study may include limitation due to lack of prior qualitative research studies, the clinical implication that can be taken from this study was; identifying psychological factors that hinder weaning success can enable the development of tailored interventions. For instance, implementing relaxation techniques, cognitive-behavioral therapy, or providing psychological support may assist patients in coping with anxiety or stress, potentially facilitating smoother weaning from the ventilator.

## Data availability

Data will be available for any reasonable request.

## CRediT authorship contribution statement

**Mohamed Gamal Elsehrawy:** Validation, Supervision, Project administration, Methodology, Investigation, Formal analysis, Data curation, Conceptualization. **Ahmad M. Saleh:** Writing – review & editing, Writing – original draft, Validation.

## Declaration of competing interest

The authors declare the following financial interests/personal relationships which may be considered as potential competing interests:Mohamed Gamal Elsehrawy reports financial support was provided by 10.13039/100009392Prince Sattam bin Abdulaziz University. If there are other authors, they declare that they have no known competing financial interests or personal relationships that could have appeared to influence the work reported in this paper.
